# Optimized SQE atomic charges for peptides accessible via a web application

**DOI:** 10.1186/s13321-021-00528-w

**Published:** 2021-06-30

**Authors:** Ondřej Schindler, Tomáš Raček, Aleksandra Maršavelski, Jaroslav Koča, Karel Berka, Radka Svobodová

**Affiliations:** 1 CEITEC-Central European Institute of Technology, Masaryk University, Kamenice 5, 602 00 Brno, Czech Republic; 2 National Centre for Biomolecular Research, Faculty of Science, Masaryk University, Kamenice 5, 625 00 Brno, Czech Republic; 3 Faculty of Informatics, Masaryk University, Botanická 68a, 602 00 Brno, Czech Republic; 4 Division of Biochemistry, Department of Chemistry, Faculty of Science, University of Zagreb, Horvatovac 102a, 10000 Zagreb, Croatia; 5 Department of Physical Chemistry, Faculty of Science, Palacký University Olomouc, 17. listopadu 1192/12, 771 46 Olomouc, Czech Republic

**Keywords:** Partial atomic charges, Parameterization, Empirical methods, Web service

## Abstract

**Background:**

Partial atomic charges find many applications in computational chemistry, chemoinformatics, bioinformatics, and nanoscience. Currently, frequently used methods for charge calculation are the Electronegativity Equalization Method (EEM), Charge Equilibration method (QEq), and Extended QEq (EQeq). They all are fast, even for large molecules, but require empirical parameters. However, even these advanced methods have limitations—e.g., their application for peptides, proteins, and other macromolecules is problematic. An empirical charge calculation method that is promising for peptides and other macromolecular systems is the Split-charge Equilibration method (SQE) and its extension SQE+q0. Unfortunately, only one parameter set is available for these methods, and their implementation is not easily accessible.

**Results:**

In this article, we present for the first time an optimized guided minimization method (optGM) for the fast parameterization of empirical charge calculation methods and compare it with the currently available guided minimization (GDMIN) method. Then, we introduce a further extension to SQE, SQE+qp, adapted for peptide datasets, and compare it with the common approaches EEM, QEq EQeq, SQE, and SQE+q0. Finally, we integrate SQE and SQE+qp into the web application Atomic Charge Calculator II (ACC II), including several parameter sets.

**Conclusion:**

The main contribution of the article is that it makes SQE methods with their parameters accessible to the users via the ACC II web application (https://acc2.ncbr.muni.cz) and also via a command-line application. Furthermore, our improvement, SQE+qp, provides an excellent solution for peptide datasets. Additionally, optGM provides comparable parameters to GDMIN in a markedly shorter time. Therefore, optGM allows us to perform parameterizations for charge calculation methods with more parameters (e.g., SQE and its extensions) using large datasets.

**Graphic Abstract:**

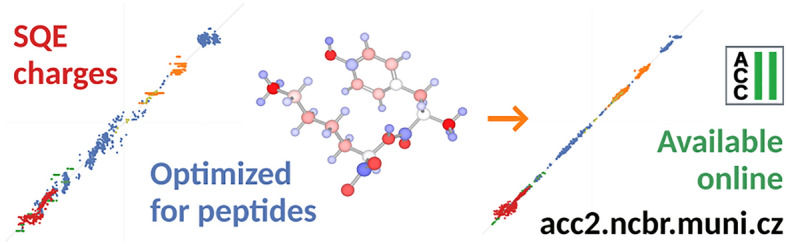

## Introduction

Partial atomic charges are real numbers assigned to individual atoms of a molecule that approximate the distribution of electron density among these atoms. Partial atomic charges find many applications in computational chemistry [1–3], chemoinformatics [4–6], bioinformatics [7, 8], and nanoscience [9, 10]. Because the charges are not physicochemical observables but a theoretical concept, many methods for their calculation have been developed. The most reliable are quantum mechanical (QM) methods, because they are calculated according to the standard definition of partial atomic charges. Specifically, they compute the distribution of electrons in orbitals (the so-called electron population of the orbitals) and divide this electron population among individual atoms via a population analysis (e.g., MPA [11, 12], NPA [13, 14]) or charge calculation scheme (e.g., ESP [15], RESP [16]). A substantial disadvantage of QM approaches is their high computational complexity, and therefore a long computational time.

Empirical charge calculation methods are faster alternatives to QM methods. They calculate charges based on common physicochemical laws (e.g., Coulomb law), but they include empirical parameters derived from values of QM charges or other tabular values or constants. Currently, frequently used empirical methods are the Electronegativity Equalization Method (EEM) [17], Charge Equilibration method (QEq) [18], and Extended QEq (EQeq) [19]. However, even these advanced and popular methods have their limitations—e.g., their application for peptides, proteins, and other homogeneous macromolecular systems (i.e., systems composed from just several types of residues) is problematic. The reason for this is that in these macromolecules, individual types of atoms (e.g., single-bonded O) have charge values that are spread over a small range (or a few small ranges), and such disproportional charge distribution is a challenge for parameterization approaches. Especially when charge differences in the whole molecule are small (no highly positive or negative atoms or ions are present), the charge ranges are tiny. However, there are promising empirical charge calculation methods: the Split-charge Equilibration method (SQE) [20] and its extension to peptides, SQE+q0 [21].

Unfortunately, implementations of these methods and their parameters are not easily accessible to the public, so their potential usage is limited.

Recently, also machine learning approaches were applied in the area of partial atomic charges computation [22–25]. However, they are primarily targeted at small heterogeneous molecules with a firm conformation. Moreover, recent approaches [24, 25] impose limits on the size of the molecule (having at most 65 atoms) which is the limitation empirical methods don’t have.

In this publication, we have reimplemented the SQE and SQE+q0 methods and compared them with other currently popular empirical approaches. Furthermore, we introduce another SQE extension, SQE+qp, adapted for peptides. An essential goal of our article is also to make SQE and SQE+qp implementation accessible for the research community via the web application Atomic Charge Calculator II (ACC II) [26], including several parameter sets. Finally, this article also presents an optimized guided minimization method (optGM) for the fast parameterization of empirical charge calculation methods.

### Description of SQE and SQE+q0 methods

#### SQE

SQE is based on the electronegativity equalization principle. However, unlike EEM or QEq, it does not perform equalization at the level of individual atoms, but switches the problem to a bond domain by defining *split-charges*, i.e., charges located on the bonds. Formally, the atomic charge on atom *i* is expressed as the sum of those split-charges on bonds that a particular atom is a part of:

$$\begin{aligned} q_i = \sum _{j \in \text {BA}(i)} p_{i, j} \end{aligned}$$

where $\text {BA}(i)$ is the set of atoms bonded to atom *i*, and $p_{i, j}$ is the split-charge on the bond $i - j$.

The SQE method written in the form of a system of linear equations is described by the equation:

$$\begin{aligned} \left( THT^T + \text {diag}(\kappa )\right) {q_{sp}} = T\chi \end{aligned}$$

where $q_{sp}$ is the vector of split-charges, *T* is the incidence matrix describing the molecular topology, $\text {diag}(\kappa )$ is the diagonal matrix with bond hardnesses, $\chi$ is the vector of atomic electronegativities, and *H* is the hardness matrix that describes the interactions between the atoms.

To reconstruct the atomic charges *q* from the split-charges, the following transformation is made:

$$\begin{aligned} q = T^Tq_{sp} \end{aligned}$$

#### SQE+q0

Since the formalism of SQE has no way of setting the total charge of a molecule or the formal charge of a particular atom, it might not be very well suited to accounting for the charged functional groups found, for example, in peptides. This shortcoming was addressed in SQE+q0 [21], an extension to SQE. SQE+q0 adds formal charges to work as initial seeds for the computation of partial atomic charges. This change is expressed in:

$$\begin{aligned} \left( THT^T + \text {diag}(\kappa )\right) q_{sp} = T(\chi - Hq_0 + \eta * q_0) \end{aligned}$$

where $q_0$ is the vector of initial formal charges, $\eta$ is the vector of atomic hardnesses, and $*$ is the element-wise product. The calculation of atomic charges is then trivially modified to:

$$\begin{aligned} {q} = T^Tq_{sp} + q_0 \end{aligned}$$

## Methods

### Description of SQE+qp method

Our new method SQE+qp replaces the formal charge $q_0$ of a SQE+q0 method with the member $q_p$, representing the initial charge of the relevant atomic type. Since the sum of the initial charges can differ from the total molecular charge, simple normalization must be performed before the actual computation. The following equation describes this normalization:

$$\begin{aligned} q_p^{norm} = q_p - \frac{1}{N}\left( 1^Tq_p - Q\right) \end{aligned},$$

where Q is the total molecular charge, and N is the number of atoms in the molecule. The values of initial charges are obtained in the process of parameterization of the SQE+qp method.

### Implementation of empirical methods for partial charge calculation

All the methods which are used in this paper are implemented as modules of ACC II. Specifically, EEM, QEq, and EQeq were already present in ACC II, and their implementations were based on the descriptions in articles [17, 18], and [19], respectively. SQE, SQE+q0, and SQE+qp were recently added to ACC II as a result of this work. SQE and SQE+q0 were implemented according to [20, 27]. The implementation of SQE+qp is based on the previous works and this article.

ACC II is freely available under the MIT license at GitHub [28]. Furthermore, all ACC II charge calculation methods can be used via a standalone command-line application [29] that enables users to integrate charge calculation methods (including SQE-like methods) into their own workflows. While the application and all the methods are implemented in C++ language to achieve the best performance, we also provide Python bindings to these methods for convenience. A short description of the methods can be found at the ACC II webpage [30].

### Parameterization of empirical methods

Several key aspects largely influence the parameterization process, namely, the differentiation of atoms (and bonds) into atomic (and bond) types, the global optimization scheme, and the design of the objective function that evaluates the parameters’ quality using several standard metrics. Note that the implementations of all the parameterization schemes mentioned in this section are a part of our internal package MACH, available freely at GitHub [31].

#### Atomic and bond types

During the parameterization, each atom is assigned a type that shares the same values of individual parameters. Multiple schemes for assigning types can be employed, from the simplest, in which an atom’s element represents the type, to more complex ones. One of the widely used approaches is to differentiate the atoms based on the element and the highest bond order of the bond they are part of [32–34]. In this text, we use the acronym HBO (highest bond order) to denote such classification (e.g., a carbon with a double bond would be C/2, an oxygen with only single bonds is O/1). The second scheme we used describes an atom’s bonded environment, i.e., all the bonded atoms (BA). Examples might be C/CCCH for a carbon connected to three other carbon atoms and one hydrogen, or O/CH for an oxygen connected to a carbon and a hydrogen.

Since SQE includes bond parameters, we must also categorize each bond. The bond type is based on the atomic types of the constituent atoms and the order of the bond.

#### Optimization scheme

We used the guided minimization (GDMIN) [35] method to parameterize all the above-mentioned empirical methods. Unfortunately, we found that GDMIN is very time-consuming for SQE-like methods, because they require parameters for bonds, which are not present in EEM, QEq, and EQeq. Moreover, this problem is amplified by the usage of BA atomic types, which allows for a greater number of potential combinations of bonded atoms. Therefore the number of parameters increases significantly. For this reason, we developed the method optGM, an improvement of GDMIN designed to reach the same or better results in a markedly shorter time. The main differences between GDMIN and optGM are:

- optGM only uses a suitable subset (i.e., a subset of molecules containing at least N atoms of each atomic type present in the original training set) of molecules in several steps of the parameterization process. Evaluation of the objective function in these steps is therefore significantly faster.
- The number of initial samples can be substantially higher (since they are only evaluated on a subset) than in the original approach developed for EEM, which has only two parameters per atomic type. A large number of initial samples is necessary to sufficiently cover the parameter space in methods with multiple atom and bond parameters.
- The number of local optimizations, which are the most time-demanding part of the parameterization, is limited to just the best candidate samples.

Further details about optGM are described in Additional file 1: Section 1.

#### Quality metrics

To be able to evaluate the quality of the parameters, quality criteria must be defined. All of them describe the correspondence between the reference QM charges $X = (x_1, \ldots , x_N)$ and the empirical charges $Y = (y_1, \ldots , y_N)$ produced as a result of the parameterization process. In this work, we use the most common quality metrics, specifically:

$\text {R}^2$ *Squared value of Pearson’s correlation coefficient*. This metric describes the linear correlation between two sets of values. Values close to 1 indicate a strong linear correlation, whereas values near zero indicate a low correlation.

$$\begin{aligned} \text {R}^2(X, Y) = \frac{\left( \sum _{i=1}^{N}(x_i-{{\overline{x}}})(y_i-{{\overline{y}}})\right) ^2}{ \sum _{i=1}^{N}\left( x_i-{\overline{x}}\right) ^2 \sum _{i=1}^{N}(y_i-{\overline{y}})^2} \end{aligned}$$

where ${\overline{x}}$ and ${\overline{y}}$ represent the mean values of the sets *X* and *Y*, respectively.

$\text {RMSD}$ *Root mean square deviation*. The lower the value of RMSD, the more similar the two sets of values are. A zero value indicates that the sets are identical.

$$\begin{aligned} \text {RMSD}(X, Y) = \displaystyle \sqrt{\frac{1}{N} \sum _{i=1}^N \left( x_i - y_i\right) ^2} \end{aligned}$$

${\text {RMSD}_{at}}$ *RMSD for atomic type*. This quantity represents the worst (i.e., the largest) value of the RMSD values computed for individual atomic types.

In this work, the values of $\text {R}^2$ and $\text {RMSD}$ are computed for each molecule and then averaged over the whole set.

#### Objective function

The evaluation of the objective function guides the steps of the global optimization method. In this paper, we used the function defined as the sum of averaged RMSD values calculated for each molecule and the average of $\text {RMSD}$ values for each atomic type.

#### Correlation graphs

**Fig. 1:**
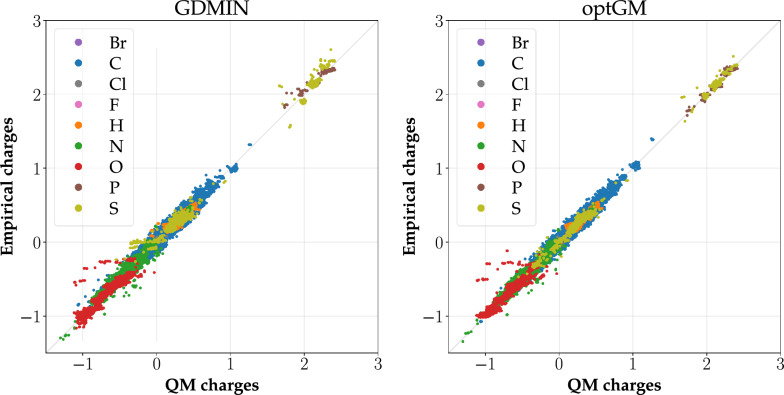
Correlation graphs for CCD_gen dataset. Empirical charges are calculated using the parameters obtained by GDMIN and by optGM

In parallel with quality metrics, a correlation between reference (QM) and empirical charges can also be evaluated using a correlation graph. The X-axis of the graph contains QM charges and the Y-axis empirical charges. Each point of the graph represents one atom and pairs its QM and empirical charges. Moreover, individual points are colored according to their atomic type. Therefore it can be directly seen which type of atoms correlates weakly. An example of a correlation graph can be found in Fig. 1.

## Results and discussions

To assess the empirical methods and the parameterization schemes described in the previous section, we devised a series of experiments. First, the choice of datasets and reference charges had to be made.

### Datasets

In this paper, we utilized three datasets of molecules, described in Table 1. The first two datasets are composed of organic molecules and were also used for the comparison and parameterization of empirical charge calculation methods in previous publications [33, 34]. DTP_small is a simple set (a low number of small-sized molecules with low variability) while CCD_gen is more complex. DTP_small contains organic molecules used as drugs; CCD_gen includes organic molecules acting as protein ligands. The last dataset, PUB_pept, was created directly for this publication. It contains small peptides obtained from the PubChem database [36]. It represents a dataset of molecules with homogeneous atomic types. The methodology of how this dataset was prepared is described in Additional file 1: Section 2.

Each dataset was divided into two subsets: a training set and a test set containing 80% and 20% of the molecules, respectively. The division was done randomly, and the stratification was included during the separation. The list of molecules that comprised the training and test set can be found in Additional file 2. For all the datasets, molecules in SDF format are provided in Additional file 3.

**Table 1:** Summary information about datasets used in this work

	Dataset		
Denotation	DTP_small	CCD_gen	PUB_pept
Source database	DTP NCI	wwPDB CCD	PubChem
Number of molecules	1,956	4,443	60
Number of atoms	62,977	204,760	2,636
Atomic types (elements and bond orders)	H/1, C/1, C/2, N/1, N/2, O/1, O/2, S/1	H/1, C/1, C/2, C/3, N/1, N/2, N/3, O/1, O/2, F/1, P/2, S/1, S/2, Cl/1, Br/1	H/1, C/1, C/2, N/1, N/2, O/1, O/2, S/1
Size of molecules (number of atoms)	6-176	3-305	20-70
The main type of molecules	Organic molecules (drug-like)	Organic molecules (ligands)	Di- and tripeptides
Source of 3D structures	Generated by CORINA		From PubChem (generated by OEOmega)
Reference to publication	[34, 37]	[34]	-

### Reference charges

The QM charge calculation approach B3LYP/6-311G/NPA was selected for calculating the QM reference charges (i.e., charges used for the parameterization and evaluation of all the compared empirical methods) on datasets DTP_small and CCD_gen. These charges were used because the combination of the B3LYP theory level, the 6-311G basis set, and NPA proved to be very suitable for parameterizing empirical charge calculation methods [4, 5, 33, 38]. For the dataset PUB_pept, the QM charge calculation approach B3LYP/6-31G*/NPA was selected. The method and the population analysis are the same as for the first two datasets, but the basis set 6-31G* was used. The reason for this is that 6-311G is too complex and not applicable for peptide molecules. The basis set 6-31G* represents a robust enough and feasible replacement, and was also often used to parameterize empirical charge calculation methods [32, 39, 40]. The QM charges for all the datasets were calculated with Gaussian 09 [41]. The files with QM partial atomic charges for molecules from all the datasets are available in Additional file 4.

### Comparison of parameterization approaches GDMIN and optGM

As the first step of our study, we proved the applicability of the optGM method. For this purpose, a parameterization of the SQE method was performed via GDMIN and optGM for training subsets of all three datasets (with HBO atomic types). The parameterization times are summarized in Table 2. Further details about the parameterization process (setup, convergence criteria) are in Additional file 5: Section 2.

**Table 2:** Comparison of GDMIN and optGM parameterization of SQE with HBO atomic types

DTP_small	${R^2}$	$\text {RMSD}$	${\text {RMSD}_{at}}$	Param. time [h:m:s]
GDMIN	0.9941	0.0256	0.0599	19:47:22
optGM	0.9945	0.0249	0.0399	0:40:07

CCD_gen	${R^2}$	$\text {RMSD}$	${\text {RMSD}_{at}}$	Param. time [h:m:s]
GDMIN	0.9934	0.0334	0.0884	40:03:11
optGM	0.9950	0.0292	0.0406	10:27:05

PUB_pept	${R^2}$	$\text {RMSD}$	${\text {RMSD}_{at}}$	Param. time [h:m:s]
GDMIN	0.9875	0.0519	0.0756	18:37:16
optGM	0.9875	0.0518	0.0746	0:04:58

This parameterization was only done for SQE, because other empirical methods have a low number of parameters; thus their parameterization is considerably less time demanding, making GDMIN sufficient for them. The HBO atomic type was chosen because it is frequently used and only creates a small number of atomic classes. Thus the calculation of parameters is markedly less time demanding than for BA atomic types, and can even be done by GDMIN in a reasonable time (a few days). Afterward, the parameters computed for each dataset were used to calculate empirical charges for this dataset (i.e., using its training subset and also using its test subset). The values of obtained empirical and reference QM charges were compared via standard metrics (i.e., ${R^2}$, $\text {RMSD}$, and ${\text {RMSD}_{at}}$). The values of these metrics for the training subsets are summarized in Table 2. Other values of quality metrics are provided in Additional file 5: Section 2. Fig. 1 also shows correlation graphs for the whole CCD_gen dataset. Other correlation graphs are in Additional file 5: Section 3.

Table 2 shows that the parameters obtained by optGM provide charges, which correlate with QM comparably or slightly better than the charges calculated using the parameters obtained by GDMIN. The metrics for the test set show the same trend. This conclusion is also confirmed by the correlation graphs (see Fig. 1).

Moreover, Table 2 shows that optGM provides results significantly faster than GDMIN. Therefore, optGM proved to be a more appropriate parameterization approach and was used for the subsequent examinations presented in this work.

### Comparison of empirical charge calculation methods

As the second step of our study, we compared SQE, SQE+q0, and the newly developed SQE+qp method with the common approaches (i.e., EEM, QEq, and EQeq). For this comparison, a parameterization of all the methods was performed via optGM on the training subsets of all three datasets. HBO atomic types were used for all the datasets. Additionally, BA atomic types were also used for the dataset PUB_pept. The reason for this is that the PUB_pept dataset is homogeneous, since its atoms are parts of amino acids. Therefore, they have only several combinations of neighboring atoms (e.g., S can only have the following atom pairs as neighbors: C and C, C and H, C and S). Because of this, BA atomic types do not divide atoms into too many groups (which could have only a small number of atoms), which would negatively affect the parameterization process. Vice-versa, DTP_small and CCD_gen are heterogeneous datasets, and BA is not appropriate for them due to the small number of samples for the individual atomic types.

In summary, four combinations of datasets and atomic types were used (see Table 3). Further details about the parameterization process are in Additional file 6: Section 1. All the obtained parameter sets are in Additional file 7.

**Table 3:** Comparison of empirical methods on training subsets

	DTP_small (HBO)			CCD_gen (HBO)		
Method	${R^2}$	$\text {RMSD}$	${\text {RMSD}_{at}}$	${R^2}$	$\text {RMSD}$	${\text {RMSD}_{at}}$
EEM	0.9728	0.0557	0.0957	0.9790	0.0599	0.1559
QEq	0.9732	0.0552	0.0956	0.9793	0.0594	0.1565
EQeq	0.9824	0.0444	0.1014	0.9839	0.0523	0.1591
SQE	0.9952	0.0233	0.0394	0.9953	0.0282	0.0414
SQE+q0	0.9924	0.0290	0.0671	0.9928	0.0350	0.0804
SQE+qp	0.9957	0.0220	0.0398	0.9962	0.0253	0.0442

	PUB_pept (HBO)			PUB_pept (BA)		
Method	${R^2}$	$\text {RMSD}$	${\text {RMSD}_{at}}$	${R^2}$	$\text {RMSD}$	${\text {RMSD}_{at}}$
EEM	0.9790	0.0672	0.0858	0.9963	0.0281	0.0527
QEq	0.9792	0.0669	0.0851	0.9950	0.0327	0.0742
EQeq	0.9831	0.0603	0.0767	0.9962	0.0285	0.0524
SQE	0.9877	0.0513	0.0760	0.9958	0.0302	0.0510
SQE+q0	0.9936	0.0373	0.0536	0.9984	0.0188	0.0414
SQE+qp	0.9968	0.0264	0.0480	0.9992	0.0133	0.0322

Afterwards, the parameters computed for each dataset and atomic types were used to calculate empirical charges for this dataset (i.e., using its training subset and its test subset).

**Fig. 2:**
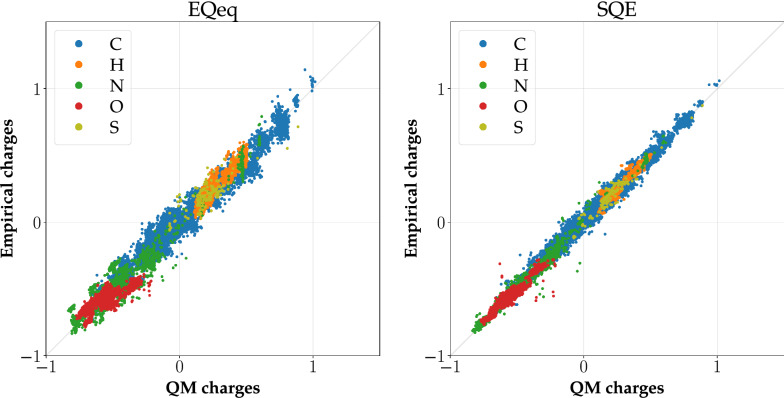
Correlation graphs for the DTP_small dataset. Empirical charges are calculated using the parameters obtained by EQeq and SQE

**Fig. 3:**
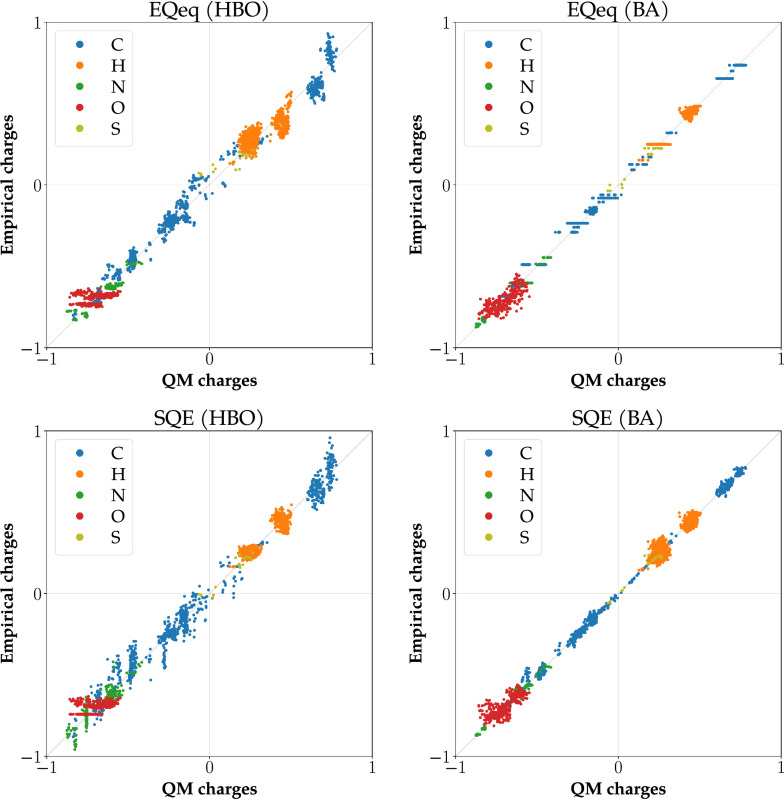
Correlation graphs for PUB_pept dataset and EQeq and SQE methods. Empirical charges in the left graphs were calculated using HBO atomic types, and in the right graphs using BA atomic types. The top graphs include empirical charges calculated by EQeq and the bottom graphs by SQE

The values of obtained empirical and reference QM charges were compared via standard metrics. The values of these metrics for the training subsets are summarized in Table 3, and the remaining values of quality metrics are in Additional file 6: Section 2. Figure 2 shows selected correlation graphs for the heterogeneous dataset CCD_gen, and Fig. 3 presents selected correlation graphs for the homogeneous dataset PUB_pept. The remaining correlation graphs are in Additional file 6: Section 3.

#### Comparison of methods for heterogeneous datasets

All methods perform well for datasets of drug-like organic molecules (see Table 3 and the high values of ${R^2}$). However, even though the quality metrics are reasonable for non-SQE approaches, the correlation graphs in Fig. 2 show examples proving that SQE describes individual atomic types better than EQeq, which proved to be the best of the traditional methods. Moreover, SQE+qp is comparable or slightly better than SQE and SQE+q0.

#### Comparison of methods for a homogeneous dataset

When considering peptides, we included both HBO and BA atomic types. Whereas the HBO types are usable for every method, the BA atomic types are not suited for EEM, QEq, and EQeq. For example, see Fig. 3, where EQeq, combined with BA atomic types, gives constant empirical charges for almost every atomic type (see X-axis parallel lines of points for most atomic types). EEM and QEq exhibit the same behavior (see correlation graphs in Supplementary information).

**Fig. 4:**
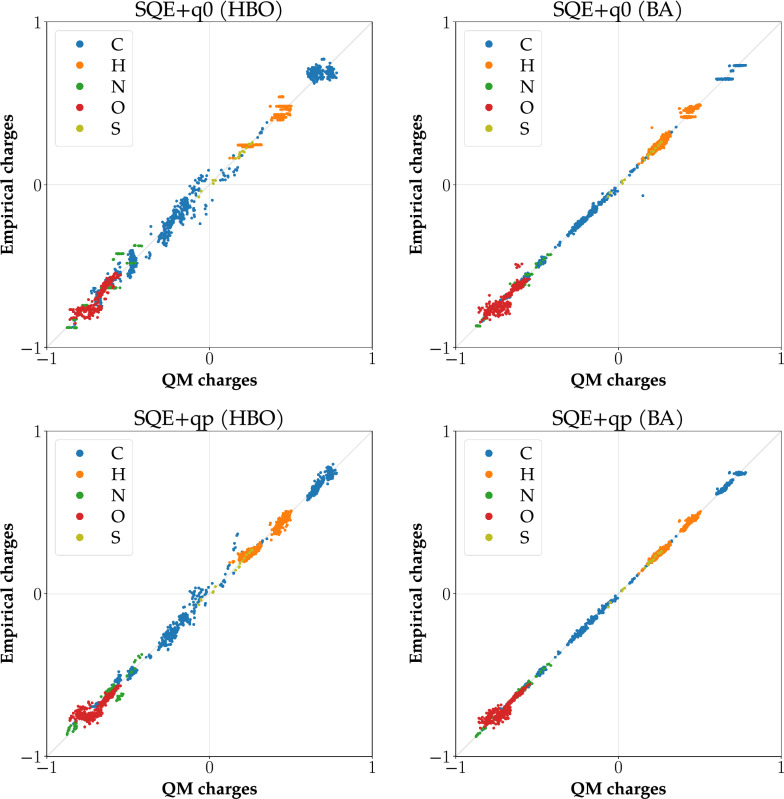
Correlation graphs for PUB_pept dataset and SQE+q0 and SQE+qp methods. Empirical charges in the left graphs were calculated using HBO atomic types, and in the right graphs using BA atomic types. The top graphs include empirical charges calculated by SQE+q0 and the bottom graphs by SQE+qp

SQE-like methods, on the other hand, can utilize the more fine-grained division of BA atomic types and generates high-quality empirical charges. However, even with these methods, we can find differences between them. See the example comparison of SQE+q0 and SQE+qp in Fig. 4. Our method SQE+qp outperforms the earlier two models for peptides and seems to be promising for other homogeneous datasets.

The complete results for all the methods and datasets are presented in Table 3.

## Conclusions

First, we developed and tested the optGM parameterization scheme. This scheme produces parameters comparable to the GDMIN method, but in a significantly shorter time. Therefore, optGM is also applicable for large datasets and charge calculation approaches with more parameters (i.e., SQE, SQE+q0, and SQE+qp). An implementation of optGM is available on GitHub.

Then, we developed the SQE+qp empirical charge calculation method and compared this method with the empirical methods EEM, QEq, EQeq, SQE, and SQE+q0. We found that for heterogeneous datasets with drug-like organic molecules, SQE-like methods performed comparably and improved upon the traditional electronegativity equalization approaches. For a homogeneous dataset with peptides, SQE+qp provided the best results and outperformed all other approaches, including SQE+q0. We also introduced a new atom classification type, BA, tailored to peptides and likely other homogeneous datasets. The combination of SQE+qp with BA atomic types proved to be an excellent solution for peptides.

The main contribution of the article is that it makes SQE, SEQ+q0 and its extension SEQ+qp together with their parameter sets accessible to the users via ACC II web application and also via a command-line application. Therefore, all these methods are now available for the broad research community for quick and precise empirical atomistic charge calculation.

## Supplementary Information

**Additional file 1.** Detailed description of parameterization process.Description of optGM parameterization scheme and preparation of PUB pept dataset.

**Additional file 2.** Division of datasets into training and test sets.Lists of IDs of molecules comprising training and test sets for each dataset.

**Additional file 3.** Datasets.Molecules for all datasets.

**Additional file 4.** QM charges.Files with QM partial atomic charges for molecules from all datasets.

**Additional file 5.** Details of GDMIN and optGM comparison.Description of procedure, values of quality metrics and correlation graphs.

**Additional file 6.** Details of empirical charge method comparison.Description of procedure, values of quality metrics and correlation graphs.

**Additional file 7.** Parameter sets.All parameter sets obtained during the comparison of empirical methods.

## Data Availability

The datasets supporting the conclusions of this article are included within the article (and its additional files).

## References

[1] Vainio MJ, Johnson MS (2007). Generating conformer ensembles using a multiobjective genetic algorithm. J Chem Inform Model.

[2] Muniz HS, Nascimento AS (2017). Towards a critical evaluation of an empirical and volume-based solvation function for ligand docking. PLoS ONE.

[3] Kritikos E, Giusti A (2020). Reactive molecular dynamics investigation of toluene oxidation under electrostatic fields: effect of the modeling of local charge distribution. J Phys Chem A.

[4] Svobodová Vařeková R, Geidl S, Ionescu C-M, Skřehota O, Kudera M, Sehnal D, Bouchal T, Abagyan R, Huber HJ, Koča J (2011). Predicting pKa values of substituted phenols from atomic charges: comparison of different quantum mechanical methods and charge distribution schemes. J Chem Inform Model.

[5] Geidl S, Svobodová Vařeková R, Bendová V, Petrusek L, Ionescu C-M, Jurka Z, Abagyan R, Koca J (2015). How does the methodology of 3D structure preparation influence the quality of pKa prediction?. J Chem Inform Model.

[6] Kumar SP, Jha PC, Jasrai YT, Pandya HA (2015). The effect of various atomic partial charge schemes to elucidate consensus activity-correlating molecular regions: a test case of diverse QSAR models. J Biomol Struct Dyn.

[7] Holliday JD, Jelfs SP, Willett P, Gedeck P (2003). Calculation of intersubstituent similarity using R-group descriptors. J Chem Inform Comp Sci.

[8] Cleves AE, Johnson SR, Jain AN (2019). Electrostatic-field and surface-shape similarity for virtual screening and pose prediction. J Comput Aided Mol Design.

[9] Chuang C-H, Porel M, Choudhury R, Burda C, Ramamurthy V (2018). Ultrafast electron transfer across a nanocapsular wall: coumarins as donors, viologen as acceptor, and octa acid capsule as the mediator. J Phys Chem B.

[10] Luo D, Wang F, Chen J, Zhang F, Yu L, Wang D, Willson RC, Yang Z, Ren Z (2018). Poly(sodium 4-styrenesulfonate) Stabilized Janus Nanosheets in Brine with Retained Amphiphilicity. Langmuir.

[11] Mulliken RS (1955). Electronic population analysis on LCAO-MO molecular wave functions. J Chem Phys.

[12] Mulliken RS (1955). Electronic population analysis on LCAO-MO molecular wave functions. II. Overlap populations, bond orders, and covalent bond energies. J Chem Phys.

[13] Reed AE, Weinhold F (1983). Natural bond orbital analysis of near-Hartree-Fock water dimer. J Chem Phys.

[14] Reed AE, Weinstock RB, Weinhold F (1985). Natural population analysis. J Chem Phys.

[15] Singh UC, Kollman PA (1984). An approach to computing electrostatic charges for molecules. J Comput Chem.

[16] Bayly CI, Cieplak P, Cornell W, Kollman PA (1993). A well-behaved electrostatic potential based method using charge restraints for deriving atomic charges: the RESP model. J Phys Chem.

[17] Mortier WJ, Ghosh SK, Shankar S (1986). Electronegativity equalization method for the calculation of atomic charges in molecules. J Am Chem Soc.

[18] Rappé AK, Goddard WA (1991). Charge equilibration for molecular dynamics simulations. J Phys Chem.

[19] Wilmer CE, Kim KC, Snurr RQ (2012). An extended charge equilibration method. J Phys Chem Lett.

[20] Nistor RA, Polihronov JG, Müser MH, Mosey NJ (2006). A generalization of the charge equilibration method for nonmetallic materials. J Chem Phys.

[21] Verstraelen T, Pauwels E, De Proft F, Van Speybroeck V, Geerlings P, Waroquier M (2012). Assessment of atomic charge models for gas-phase computations on polypeptides. J Chem Theory Comput.

[22] Bleiziffer P, Schaller K, Riniker S (2018). Machine learning of partial charges derived from high-quality quantum-mechanical calculations. J Chem Inform Model.

[23] Martin R, Heider D (2019). ContraDRG:automatic partial charge prediction by machine learning. Front Genet.

[24] Wang J, Cao D, Tang C, Chen X, Sun H, Hou T (2020). Fast and accurate prediction of partial charges using Atom-Path-Descriptor-based machine learning. Bioinformatics.

[25] Wang J, Cao D, Tang C, Xu L, He Q, Yang B, Chen X, Sun H, Hou T (2021). DeepAtomicCharge: a new graph convolutional network-based architecture for accurate prediction of atomic charges. Brief Bioinform.

[26] Raček T, Schindler O, Toušek D, Horský V, Berka K, Koča J, Svobodová R (2020). Atomic Charge Calculator II: web-based tool for the calculation of partial atomic charges. Nucleic Acids Re.

[27] Verstraelen T, Ayers PW, van Speybroeck V, Waroquier M (2013). ACKS2: Atom-condensed Kohn-Sham DFT approximated to second order. J Chem Phys.

[28] Raček T (2021) krab1k/AtomicChargeCalculator2. https://github.com/krab1k/AtomicChargeCalculator2 Accessed 8 Mar 2021

[29] Raček T (2021) krab1k/ChargeFW2. https://github.com/krab1k/ChargeFW2 Accessed 8 Mar 2021

[30] Raček T (2021) Short description of the methods. https://acc2.ncbr.muni.cz/static/methods.pdf Accessed 8 Mar 2021

[31] Schindler O (2021) dargen3/MACH. https://github.com/dargen3/MACH Accessed 8 Mar 2021

[32] Ouyang Y, Ye F, Liang Y (2009). A modified electronegativity equalization method for fast and accurate calculation of atomic charges in large biological molecules. Phys Chem Chem Phys.

[33] Geidl S, Bouchal T, Raček T, Svobodová Vařeková R, Hejret V, Křenek A, Abagyan R, Koča J (2015). High-quality and universal empirical atomic charges for chemoinformatics applications. J Cheminform.

[34] Raček T, Pazúriková J, Svobodová Vařeková R, Geidl S, Křenek A, Falginella FL, Horský V, Hejret V, Koča J (2016). NEEMP: software for validation, accurate calculation and fast parameterization of EEM charges. J Cheminform.

[35] Pazúriková J, Křenek A, Matyska L (2016) Guided optimization method for fast and accurate atomic charges computation. In: Proceedings of the 2016 European simulation and modelling conference, EUROSIS - ETI, Ghent, Belgium, pp 267–274

[36] Kim S, Chen J, Cheng T, Gindulyte A, He J, He S, Li Q, Shoemaker BA, Thiessen PA, Yu B, Zaslavsky L, Zhang J, Bolton EE (2019). PubChem 2019 update: improved access to chemical data. Nucleic Acids Res.

[37] Svobodová Vařeková R, Jiroušková Z, Vaněk J, Suchomel Š, Koča J (2007). Electronegativity equalization method: parameterization and validation for large sets of organic, organohalogene and organometal molecule. Int J Mol Sci.

[38] Ionescu C-M, Geidl S, Svobodová Vařeková R, Koča J (2013). Rapid calculation of accurate atomic charges for proteins via the electronegativity equalization method. J Chem Inform Model.

[39] Bultinck P, Langenaeker W, Lahorte P, De Proft F, Geerlings P, Van Alsenoy C, Tollenaere JP (2002). The electronegativity equalization method II: Applicability of different atomic charge schemes. J Phys Chem A.

[40] Bultinck P, Vanholme R, Popelier PLA, De Proft F, Geerlings P (2004). High-speed calculation of aim charges through the electronegativity equalization method. J Phys Chem A.

[41] Frisch MJ, Trucks GW, Schlegel HB, Scuseria GE, Robb MA, Cheeseman JR, Scalmani G, Barone V, Petersson GA, Nakatsuji H, Li X, Caricato M, Marenich AV, Bloino J, Janesko BG, Gomperts R, Mennucci B, Hratchian HP, Ortiz JV. Izmaylov AF, Sonnenberg JL, Williams-Young D, Ding F, Lipparini F, Egidi F, Goings J, Peng B, Petrone A, Henderson T, Ranasinghe D, Zakrzewski VG, Gao J, Rega N, Zheng G, Liang W, Hada M, Ehara M, Toyota K, Fukuda R, Hasegawa J, Ishida M, Nakajima T, Honda Y, Kitao O, Nakai H, Vreven T, Throssell K, Montgomery JA Jr, Peralta JE, Ogliaro F, Bearpark MJ, Heyd JJ, Brothers EN, Kudin KN, Staroverov VN, Keith TA, Kobayashi R, Normand J, Raghavachari K, Rendell AP, Burant JC, Iyengar SS, Tomasi J, Cossi M, Millam JM, Klene M, Adamo C, Cammi R, Ochterski JW. Martin RL, Morokuma K, Farkas O, Foresman JB, Fox DJ (2016) Gaussian 16 Revision B.01

