# Correction to: Optimized SQE atomic charges for peptides accessible via a web application

**DOI:** 10.1186/s13321-021-00531-1

**Published:** 2021-07-14

**Authors:** Ondřej Schindler, Tomáš Raček, Aleksandra Maršavelski, Jaroslav Koča, Karel Berka, Radka Svobodová

**Affiliations:** 1 CEITEC-Central European Institute of Technology, Masaryk University, Kamenice 5, 602 00 Brno, Czech Republic; 2 National Centre for Biomolecular Research, Faculty of Science, Masaryk University, Kamenice 5, 625 00 Brno, Czech Republic; 3 Faculty of Informatics, Masaryk University, Botanická 68a, 602 00 Brno, Czech Republic; 4 Division of Biochemistry, Department of Chemistry, Faculty of Science, University of Zagreb, Horvatovac 102a, 10000 Zagreb, Croatia; 5 Department of Physical Chemistry, Faculty of Science, Palacký University Olomouc, 17, listopadu 1192/12, 771 46 Olomouc, Czech Republic

## Correction to: J Cheminform (2021) 13:45 10.1186/s13321-021-00528-w

It was highlighted that in the original article [1] the heading of Table 2 was misaligned. This Correction article shows the correct Table 2. The original article has been updated.

**Table 2:** Comparison of GDMIN and optGM parameterization of SQE with HBO atomic types

DTP_small	$\mathrm {R}^{\mathrm{2}}$	$\mathrm {RMSD}$	$\mathrm {RMSD}_{\mathrm {at}}$	Param. time [h:m:s]
GDMIN	0.9941	0.0256	0.0599	19:47:22
optGM	0.9945	0.0249	0.0399	0:40:07

CCD_gen	$\mathrm {R}^{\mathrm{2}}$	$\mathrm {RMSD}$	$\mathrm {RMSD}_{\mathrm{at}}$	Param. time [h:m:s]
GDMIN	0.9934	0.0334	0.0884	40:03:11
optGM	0.9950	0.0292	0.0406	10:27:05

PUB_pept	$\mathrm {R}^{\mathrm{2}}$	$\mathrm {RMSD}$	$\mathrm {RMSD}_{\mathrm{at}}$	Param. time [h:m:s]
GDMIN	0.9875	0.0519	0.0756	18:37:16
optGM	0.9875	0.0518	0.0746	0:04:58
