# Supplementary material for: Optimized SQE atomic charges for peptides accessible via a web application
Source: J Cheminform. 2021 Jun 30;13:45. doi: 10.1186/s13321-021-00528-w (PMC8243439; doi:10.1186/s13321-021-00528-w)
Supplement: Supplementary file 1 — Additional file 1. Detailed description of parameterization process.Description of optGM parameterization scheme and preparation of PUB pept dataset. [file 13321_2021_528_MOESM1_ESM.pdf]

Additional file 1

1 Optimized guided minimization (optGM)

Optimized guided minimization is optimization scheme based on the GDMIN method. The algorithm internally defines a subset of the training set, which is used to speed up the computation in multiple stages. In particular, *subset*(N) is a set of molecules, which contains at least N atoms of each atomic type that is present in the whole set.

The general idea is to use the subset first to get the some reasonable starting point for the next stage, which utilizes the whole training set. The specific procedure is as follows:

1. Sample the parameter space using the Latin hypercube sampling.
2. Quickly evaluate the samples using *subset*(*1*).
3. Select 300 best samples.
4. Evaluate these samples on the whole training set.
5. Run local minimization for the 3 best candidate samples using *subset*(*5*).
6. Evaluate minimized individuals using the whole set and select the best one.
7. Run local minimization of the best one using the whole training set.

2 Preparation of PUB_pept dataset

All 3D conformers are downloaded as SDF files from the PubChem database, where they were generated by OEOmega. All structures are additionally manually prepared in Chimera as zwitterions (charges are assigned at pH 7) by removing hydrogen from the C-terminal α-carboxyl group and adding hydrogens to the N-terminal α-amino group. Peptides were chosen to contain all possible proteinogenic amino acids and to cover the range of integer charges, from -2, over neutral to +2. The complete list of structures is in Table 1.

Name	PubChem CID
Methionyl-valine	7010520
Cysteinyl-lysine	16122517
Arginyl-proline	151003
Glutamyl-tryptophan	100094
Isoleucyl-serine	14426033
Lysyl-leucine	4682588
Glycyl-aspartic acid	97363
Aspartyl-phenylalanine	93078
Alanyl-threonine	6427004
Histidyl-asparagine	9860193
Glutaminyl-tyrosine	9972600
Seryl-arginine	9856515
Phenylalanyl-cysteine	18218231
Leucyl-methionine	118276
Threonyl-glycine	7010576
Asparaginyl-histidine	18218181
Valyl-tryptophan	168182
Tyrosyl-lysine	19786231
Prolyl-glutamic acid	194260
Tryptophyl-arginine	21689254
Alanyl-glycine	6998029
Asparaginyl-lysine	21451266
Aspartyl-alanine	4677380
Aspartyl-glutamate	151403
Cysteinyl-Alanine	20574727
L-Cystine	67678
Leucyl-tyrosine	273262
Lysyl-serine	151428
Seryl-Valine	7020159

Name	PubChem CID
Lysyl-tyrosyl-tryptophan	132605043
Alanyl-lysyl-arginine	18218442
Threonyl-tyrosyl-glycine	11186822
Phenylalanyl-cysteinyl-methionine	145457179
Leucyl-cysteinyl-histidine	145456383
Arginyl-phenylalanyl-glycine	53855445
Methionyl-glutamyl-aspartic acid	60163650
Aspartyl-phenylalanyl-proline	145454528
Valyl-asparaginyl-leucine	145458852
Glycyl-glutaminyl-arginine	18221000
Tryptophanyl-alanyl-phenylalanine	145458266
Cysteinyl-prolyl-asparagine	145454856
Histidyl-aspartyl-alanine	145455777
Isoleucyl-methionyl-threonine	145456228
Tyrosyl-arginyl-serine	145458565
Prolyl-leucyl-lysine	18223611
Seryl-tryptophanyl-glutamic acid	145457897
Glutamyl-seryl-glycine	145455491
Asparaginyl-arginyl-histidine	145454019
Valyl-glutamyl-glutamine	145458915
Alanyl-glutamyl-lysine	10337675
Arginyl-alanyl-lysine	18218605
Aspartyl-glutamyl-proline	145454428
Aspartyl-methionyl-glutamic acid	145454508
L-Cystinyl-bis-L-valine	92274598
Glutamyl-phenylalanyl-tyrosine	14389323
Lysyl aspartyl isoleucine	90478376
Lysyl-histidyl-aspartic acid	145456667
Methionyl-prolyl-alanine	138319092
Phenylalanyl-threonyl-tyrosine	131801092
Phenylalanyl-valyl-arginine	10342092

Table 1: List of dipeptides and tripeptides in the PUB_pept dataset.
