# Supplementary material for: Optimized SQE atomic charges for peptides accessible via a web application
Source: J Cheminform. 2021 Jun 30;13:45. doi: 10.1186/s13321-021-00528-w (PMC8243439; doi:10.1186/s13321-021-00528-w)
Supplement: Supplementary file 5 — Additional file 5. Details of GDMIN and optGM comparison.Description of procedure, values of quality metrics and correlation graphs. [file 13321_2021_528_MOESM5_ESM.pdf]

Additional file 5

1 Comparison of GDMIN and optGM procedure

To compare GDMIN with optGM we setup the following procedure. We run each parameterizations scheme for SQE method on all datasets, i.e., DTP_small, CCD_gen and PUB_pept, using multiple numbers of initial samples. In particular, we started with 1000, and doubled the number in each new parameterization. The procedure ended if we reached 8192000 initial samples, or the individual parameterization took more than 50 hours to finish. From these results, we selected the best parameters according to the common quality criteria and compared the time that each scheme used.

2 The values of quality criteria for comparison of GDMIN and optGM

The best quality parameters for each dataset and method are written in bold.

	Training set			Test set			
Samples	R^2	RMSD	$RMSD_{at}$	R^2	RMSD	$RMSD_{at}$	Time
1000	0.9932	0.0277	0.0664	0.9934	0.0275	0.0505	4:41:49
2000	0.9937	0.0264	0.0649	0.9939	0.0265	0.0518	9:23:56
**4000**	**0.9941**	**0.0256**	**0.0599**	**0.9943**	**0.0255**	**0.0501**	**19:47:22**
8000	0.9941	0.0257	0.0476	0.9943	0.0257	0.0546	37:39:45

Table 1: GDMIN, DTP_small

	Training set			Test set			
Samples	R^2	RMSD	$RMSD_{at}$	R^2	RMSD	$RMSD_{at}$	Time
1000	0.9888	0.9887	0.0437	0.0431	0.1033	0.1160	18:20:08
**2000**	**0.9934**	**0.9933**	**0.0334**	**0.0331**	**0.0884**	**0.1087**	**40:03:11**

Table 2: GDMIN, CCD_gen

Samples	Training set R^2	RMSD	$RMSD_{at}$	Test set R^2	RMSD	$RMSD_{at}$	Time
1000	0.9732	0.0758	0.1244	0.9723	0.0784	0.127	0:30:59
2000	0.9861	0.0547	0.0751	0.9838	0.0599	0.1249	1:04:34
4000	0.9850	0.0568	0.0811	0.9818	0.0635	0.1003	16:02:46
8000	0.9872	0.0525	0.0755	0.9858	0.0562	0.088	3:59:08
16000	0.9870	0.0527	0.0749	0.9858	0.0561	0.0871	6:41:19
32000	0.9868	0.0532	0.0751	0.9852	0.0573	0.0876	9:22:34
64000	0.9874	0.0519	0.0748	0.9861	0.0556	0.0872	8:34:44
128000	0.9871	0.0525	0.0748	0.9860	0.0556	0.0879	9:57:46
256000	0.9874	0.0520	0.0748	0.9861	0.0555	0.0887	9:54:33
512000	0.9874	0.0521	0.0758	0.9857	0.0563	0.0887	10:49:55
1024000	0.9872	0.0523	0.0758	0.9860	0.0558	0.0898	11:35:07
2048000	0.9871	0.0526	0.075	0.9858	0.0561	0.0871	13:27:53
**4096000**	**0.9875**	**0.0519**	**0.0756**	**0.9862**	**0.0552**	**0.0882**	**18:37:16**
8192000	0.9871	0.0527	0.0748	0.9856	0.0564	0.0872	26:48:36

Table 3: GDMIN, PUB_pept

Samples	Training set R^2	RMSD	$RMSD_{at}$	Test set R^2	RMSD	$RMSD_{at}$	Time
1000	0.9943	0.0253	0.0416	0.9946	0.0250	0.0436	0:31:13
2000	0.9944	0.0251	0.0404	0.9947	0.0247	0.0422	0:32:07
4000	0.9944	0.0251	0.0401	0.9947	0.0247	0.0409	0:34:28
8000	0.9937	0.0266	0.0441	0.9941	0.0261	0.0450	0:32:26
16000	0.9944	0.0250	0.0403	0.9948	0.0246	0.0401	0:41:41
**32000**	**0.9945**	**0.0249**	**0.0399**	**0.9949**	**0.0244**	**0.0401**	**0:40:07**
64000	0.9943	0.0252	0.0406	0.9947	0.0248	0.0419	0:33:27
128000	0.9944	0.0250	0.0400	0.9948	0.0246	0.0394	0:43:45
256000	0.9945	0.0249	0.0400	0.9948	0.0245	0.0400	0:59:04
512000	0.9946	0.0247	0.0410	0.9949	0.0243	0.0406	1:03:06
1024000	0.9943	0.0252	0.0445	0.9946	0.0250	0.0466	0:58:38
2048000	0.9944	0.0252	0.0403	0.9947	0.0247	0.0397	1:23:42
4096000	0.9945	0.0248	0.0410	0.9949	0.0244	0.0402	2:36:31
8192000	0.9944	0.0251	0.0407	0.9947	0.0247	0.0411	4:33:03

Table 4: optGM, DTP_small

Samples	Training set R^2	RMSD	$RMSD_{at}$	Test set R^2	RMSD	$RMSD_{at}$	Time
1000	0.9946	0.0303	0.0439	0.9945	0.0300	0.0440	3:56:03
2000	0.9949	0.0295	0.0432	0.9947	0.0293	0.0575	4:34:51
4000	0.9949	0.0295	0.0415	0.9948	0.0292	0.0522	5:22:46
8000	0.9949	0.0293	0.0422	0.9948	0.0291	0.0594	4:04:27
16000	0.9949	0.0295	0.0437	0.9948	0.0293	0.0581	3:47:50
32000	0.9946	0.0303	0.0439	0.9945	0.0300	0.0436	4:25:54
64000	0.9945	0.0306	0.0475	0.9943	0.0304	0.0711	3:39:32
128000	0.9944	0.0307	0.0482	0.9943	0.0306	0.0717	3:30:11
256000	0.9946	0.0303	0.0441	0.9945	0.0300	0.0437	5:41:24
512000	0.9950	0.0293	0.0451	0.9948	0.0290	0.0578	5:06:43
1024000	0.9945	0.0306	0.0471	0.9943	0.0304	0.0706	4:55:31
2048000	0.9943	0.0311	0.0440	0.9942	0.0309	0.0594	5:30:41
**4096000**	**0.9950**	**0.0292**	**0.0406**	**0.9949**	**0.0290**	**0.0542**	**10:27:05**
8192000	0.9945	0.0305	0.0466	0.9944	0.0303	0.0695	14:07:21

Table 5: optGM, CCD_gen

Samples	Training set R^2	RMSD	$RMSD_{at}$	Test set R^2	RMSD	$RMSD_{at}$	Time
1000	0.9819	0.0623	0.0856	0.9751	0.0743	0.1240	0:04:00
2000	0.9799	0.0656	0.0805	0.9769	0.0716	0.0930	0:03:50
4000	0.9792	0.0668	0.0959	0.9775	0.0706	0.0975	0:03:59
8000	0.9754	0.0726	0.1082	0.9746	0.0750	0.1161	0:03:15
**16000**	**0.9875**	**0.0518**	**0.0746**	**0.9860**	**0.0556**	**0.0873**	**0:04:58**
32000	0.9787	0.0676	0.1157	0.9767	0.0718	0.1257	0:06:04
64000	0.9757	0.0722	0.1099	0.9747	0.0748	0.1183	0:04:54
128000	0.9839	0.0588	0.0774	0.9826	0.0620	0.0956	0:08:21
256000	0.9798	0.0658	0.0793	0.9765	0.0721	0.0920	0:11:55
512000	0.9873	0.0522	0.0740	0.9860	0.0557	0.0859	0:18:26
1024000	0.9789	0.0672	0.0950	0.9771	0.0712	0.0937	0:33:07
2048000	0.9791	0.0670	0.0837	0.9764	0.0723	0.0933	1:02:22
4096000	0.9841	0.0583	0.0778	0.9828	0.0617	0.0956	2:01:20
8192000	0.9799	0.0656	0.0806	0.9769	0.0716	0.0927	3:53:41

Table 6: optGM, PUB_pept

3 Correlation graphs from the comparison of parameterization approaches GDMIN and optGM

Following figures compares the best results for each dataset.

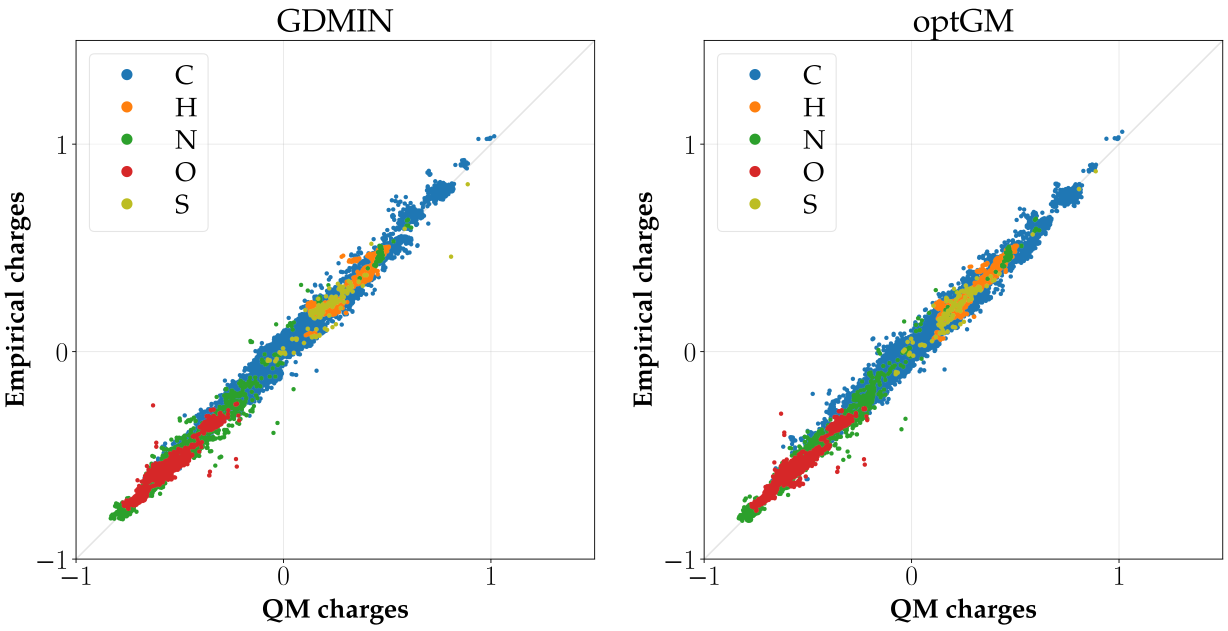

Figure 1: DTP_small

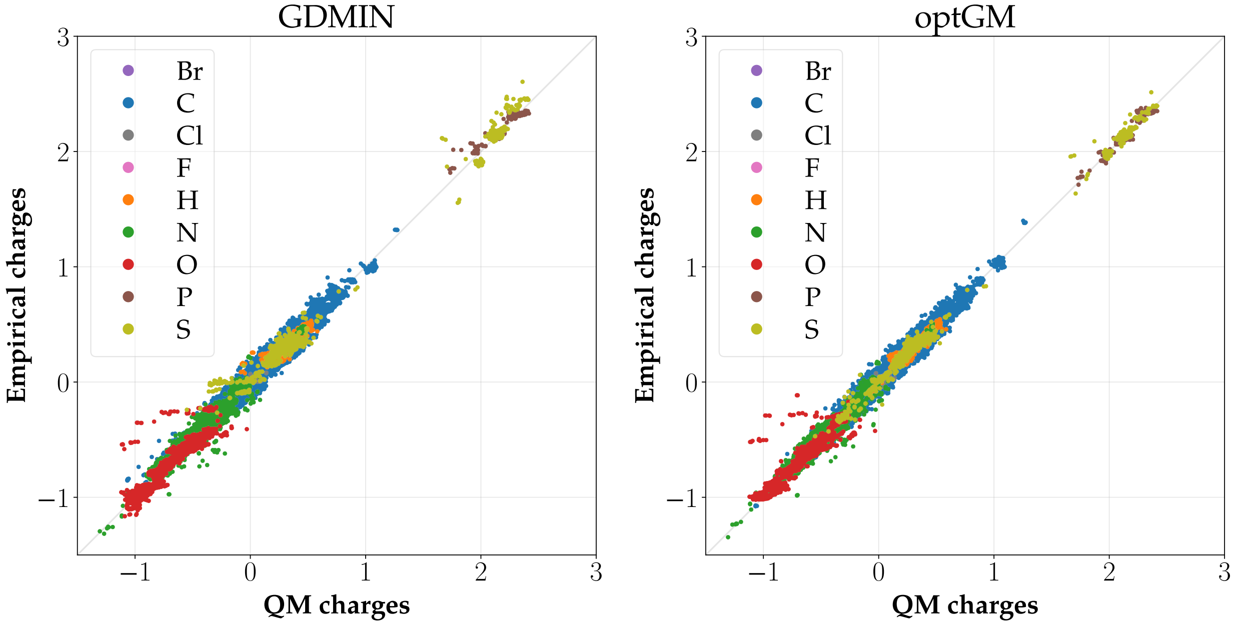

Figure 2: CCD_gen

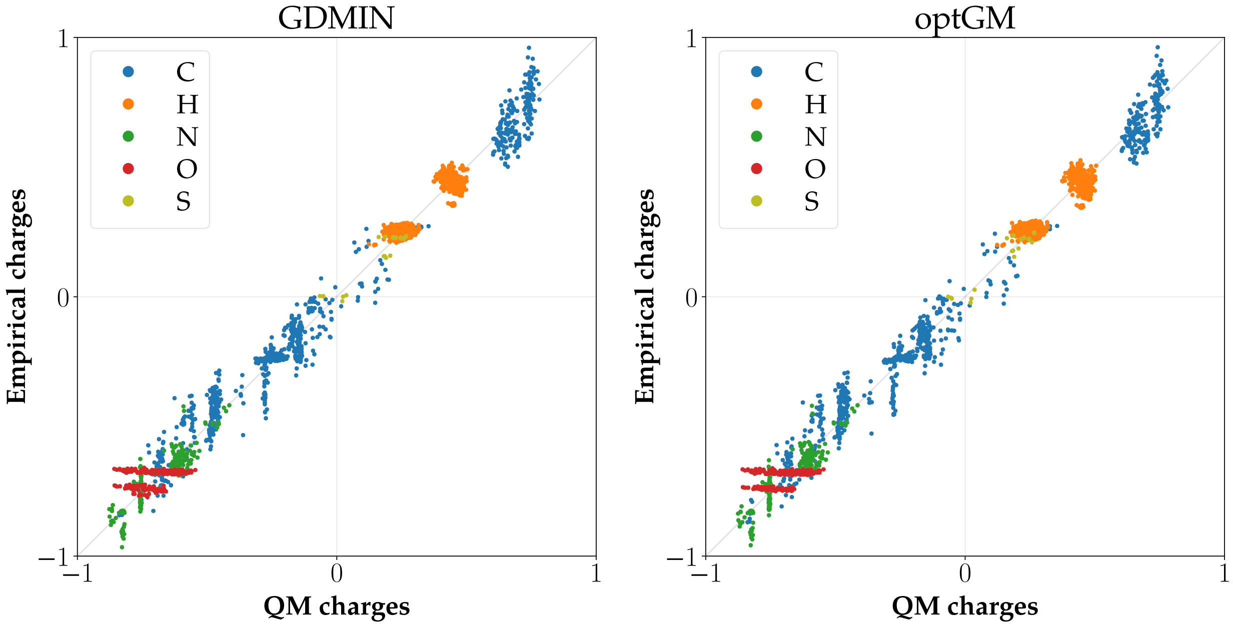

Figure 3: PUB_pept
