# Supplementary material for: Optimized SQE atomic charges for peptides accessible via a web application
Source: J Cheminform. 2021 Jun 30;13:45. doi: 10.1186/s13321-021-00528-w (PMC8243439; doi:10.1186/s13321-021-00528-w)
Supplement: Supplementary file 6 — Additional file 6. Details of empirical charge method comparison.Description of procedure, values of quality metrics and correlation graphs. [file 13321_2021_528_MOESM6_ESM.pdf]

Additional file 6

1 Comparison of empirical methods procedure

We performed the parameterization for all combination of methods and datasets, i.e., EEM, QEq, EQeq, SQE, SQE+q0, and SQE+qp and datasets DTP_small, CCD_gen, and PUB_pept. Each combination was run five times with different seeds (as there is a randomness in the initial sampling of the parameter space). The best parameters were selected for each combination and are the presented in the main article. Note that we used optGM with 10,000 initial samples.

2 The values of quality criteria for comparison of empirical methods

Since the parameterization includes randomness in the procedure, we ran each combination five times. The best quality parameters for each dataset and method are written in bold.

		Training set			Test set			
Method	Seed	R^2	RMSD	$RMSD_{at}$	R^2	RMSD	$RMSD_{at}$	Time
	**1**	**0.9728**	**0.0557**	**0.0957**	**0.9725**	**0.0569**	**0.0987**	**0:02:49**
	2	0.9728	0.0557	0.0957	0.9725	0.0569	0.0987	0:02:51
EEM	3	0.9728	0.0557	0.0957	0.9725	0.0569	0.0987	0:02:41
	4	0.9728	0.0557	0.0957	0.9725	0.0569	0.0987	0:02:40
	5	0.9728	0.0557	0.0957	0.9725	0.0569	0.0987	0:02:22
	**1**	**0.9732**	**0.0552**	**0.0956**	**0.9729**	**0.0564**	**0.0987**	**0:03:39**
	2	0.9732	0.0552	0.0956	0.9729	0.0564	0.0987	0:03:18
QEq	3	0.9732	0.0552	0.0956	0.9729	0.0564	0.0987	0:03:35
	4	0.9732	0.0552	0.0956	0.9729	0.0564	0.0987	0:03:17
	5	0.9732	0.0552	0.0956	0.9729	0.0564	0.0987	0:03:15
	**1**	**0.9824**	**0.0444**	**0.1014**	**0.9824**	**0.0451**	**0.1025**	**0:03:01**
	2	0.9824	0.0444	0.1014	0.9824	0.0451	0.1025	0:03:25
EQeq	3	0.9824	0.0444	0.1014	0.9824	0.0451	0.1025	0:03:16
	4	0.9824	0.0444	0.1014	0.9824	0.0451	0.1025	0:03:04
	5	0.9824	0.0444	0.1014	0.9824	0.0451	0.1025	0:03:19
	**1**	**0.9952**	**0.0233**	**0.0394**	**0.9954**	**0.0231**	**0.0399**	**0:53:56**
	2	0.9952	0.0233	0.0394	0.9954	0.0231	0.0399	0:53:57
SQE	3	0.9952	0.0233	0.0394	0.9954	0.0231	0.0399	0:50:58
	4	0.9952	0.0233	0.0395	0.9954	0.0231	0.0399	0:52:00
	5	0.9883	0.0363	0.0618	0.9882	0.0370	0.0641	1:20:14
	1	0.9902	0.0332	0.1363	0.9912	0.0318	0.1206	1:30:15
	2	0.9900	0.0336	0.1329	0.9908	0.0326	0.1205	1:39:06
SQE+q0	3	0.9900	0.0335	0.1378	0.9910	0.0321	0.1209	1:45:25
	4	0.9874	0.0375	0.0709	0.9870	0.0388	0.0747	1:22:12
	**5**	**0.9924**	**0.0290**	**0.0671**	**0.9926**	**0.0293**	**0.0707**	**2:02:50**
	**1**	**0.9957**	**0.0220**	**0.0398**	**0.9960**	**0.0215**	**0.0412**	**1:07:30**
	2	0.9957	0.0220	0.0398	0.9960	0.0215	0.0412	1:11:01
SQE+qp	3	0.9957	0.0220	0.0398	0.9960	0.0215	0.0412	1:06:21
	4	0.9957	0.0220	0.0398	0.9960	0.0215	0.0412	1:16:03
	5	0.9957	0.0220	0.0398	0.9960	0.0215	0.0412	1:10:12

Table 1: DTP_small

Method	Seed	Training set R^2	RMSD	$RMSD_{at}$	Test set R^2	RMSD	$RMSD_{at}$	Time
EEM	**1**	**0.9790**	**0.0599**	**0.1559**	**0.9791**	**0.0586**	**0.1734**	**0:14:12**
	2	0.9790	0.0599	0.1559	0.9791	0.0586	0.1734	0:15:00
	3	0.9790	0.0599	0.1559	0.9791	0.0586	0.1734	0:11:51
	4	0.9790	0.0599	0.1559	0.9791	0.0586	0.1734	0:14:16
	5	0.9790	0.0599	0.1559	0.9791	0.0586	0.1734	0:17:00
QEq	**1**	**0.9793**	**0.0594**	**0.1565**	**0.9794**	**0.0582**	**0.1738**	**0:35:19**
	2	0.9793	0.0594	0.1565	0.9794	0.0582	0.1738	0:39:52
	3	0.9793	0.0594	0.1565	0.9794	0.0582	0.1738	0:38:58
	4	0.9793	0.0594	0.1565	0.9794	0.0582	0.1738	0:38:54
	5	0.9793	0.0594	0.1565	0.9794	0.0582	0.1738	0:37:58
EQeq	**1**	**0.9839**	**0.0523**	**0.1591**	**0.9836**	**0.0518**	**0.1672**	**0:31:45**
	2	0.9806	0.0576	0.1585	0.9806	0.0565	0.1756	0:22:57
	3	0.9839	0.0523	0.1591	0.9836	0.0518	0.1672	0:32:58
	4	0.9806	0.0576	0.1585	0.9806	0.0565	0.1756	0:23:56
	5	0.9839	0.0523	0.1591	0.9836	0.0518	0.1672	0:32:59
SQE	1	0.9953	0.0283	0.0410	0.9952	0.0280	0.0477	17:17:16
	2	0.9953	0.0283	0.0408	0.9952	0.0280	0.0474	15:37:59
	3	0.9953	0.0283	0.0408	0.9952	0.0281	0.0474	17:37:23
	4	0.9953	0.0283	0.0412	0.9952	0.0280	0.0478	16:23:03
	**5**	**0.9953**	**0.0282**	**0.0414**	**0.9952**	**0.0279**	**0.0481**	**11:35:18**
SQE+q0	1	0.9922	0.0363	0.0784	0.9920	0.0360	0.0692	24:42:39
	2	0.9928	0.0351	0.0766	0.9924	0.0351	0.0681	21:02:14
	3	0.9928	0.0351	0.0786	0.9924	0.0351	0.07	28:19:13
	**4**	**0.9928**	**0.0350**	**0.0804**	**0.9926**	**0.0349**	**0.0726**	**20:29:19**
	5	0.9918	0.0374	0.0747	0.9912	0.0379	0.1042	23:16:55
SQE+qp	1	0.9962	0.0253	0.0442	0.9961	0.0254	0.0542	19:55:45
	**2**	**0.9962**	**0.0253**	**0.0442**	**0.9961**	**0.0253**	**0.0544**	**11:14:25**
	3	0.9962	0.0254	0.0440	0.9960	0.0254	0.0545	15:29:12
	4	0.9962	0.0253	0.0443	0.9961	0.0254	0.0543	18:22:29
	5	0.9962	0.0254	0.0441	0.9960	0.0254	0.0541	19:29:30

Table 2: CCD_gen

Method	Seed	Training set R^2	Training set RMSD	Training set $RMSD_{at}$	Test set R^2	Test set RMSD	Test set $RMSD_{at}$	Time
EEM	1	0.9790	0.0672	0.0862	0.9794	0.0675	0.0932	0:00:54
	2	0.9790	0.0672	0.0862	0.9794	0.0675	0.0931	0:00:52
	3	0.9789	0.0673	0.0877	0.9792	0.0678	0.0934	0:01:09
	**4**	**0.9790**	**0.0672**	**0.0858**	**0.9795**	**0.0674**	**0.0929**	**0:00:54**
	5	0.9790	0.0672	0.0862	0.9794	0.0675	0.0931	0:00:59
QEq	**1**	**0.9792**	**0.0669**	**0.0851**	**0.9796**	**0.0671**	**0.0990**	**0:01:00**
	2	0.9792	0.0669	0.0851	0.9796	0.0671	0.0989	0:01:01
	3	0.9792	0.0669	0.0851	0.9796	0.0671	0.0989	0:01:00
	4	0.9792	0.0669	0.0851	0.9796	0.0671	0.0989	0:00:58
	5	0.9792	0.0669	0.0851	0.9796	0.0671	0.0989	0:00:57
EQeq	**1**	**0.9831**	**0.0603**	**0.0767**	**0.9826**	**0.0620**	**0.1386**	**0:00:59**
	2	0.9831	0.0603	0.0767	0.9826	0.0620	0.1386	0:00:58
	3	0.9831	0.0603	0.0767	0.9826	0.0620	0.1386	0:01:02
	4	0.9831	0.0603	0.0767	0.9826	0.0620	0.1386	0:01:02
	5	0.9831	0.0603	0.0767	0.9826	0.0620	0.1386	0:01:03
SQE	1	0.9799	0.0656	0.0817	0.9769	0.0716	0.0950	0:09:08
	2	0.9838	0.0590	0.0777	0.9823	0.0627	0.0958	0:06:47
	**3**	**0.9877**	**0.0513**	**0.0760**	**0.9862**	**0.0553**	**0.0911**	**0:09:13**
	4	0.9820	0.0621	0.0867	0.9758	0.0732	0.1232	0:05:45
	5	0.9846	0.0575	0.0771	0.9829	0.0616	0.0953	0:09:08
SQE+q0	1	0.9926	0.0397	0.0652	0.9946	0.0346	0.0581	0:08:24
	2	0.9934	0.0377	0.0522	0.9944	0.0351	0.0586	0:08:55
	3	0.9928	0.0394	0.0679	0.9946	0.0346	0.0584	0:07:47
	4	0.9928	0.0393	0.0630	0.9942	0.0358	0.0585	0:08:48
	**5**	**0.9936**	**0.0373**	**0.0536**	**0.9946**	**0.0344**	**0.0587**	**0:10:16**
SQE+qp	1	0.9967	0.0264	0.0478	0.9973	0.0244	0.0504	0:14:27
	**2**	**0.9968**	**0.0264**	**0.0480**	**0.9973**	**0.0243**	**0.0498**	**0:13:19**
	3	0.9967	0.0264	0.0478	0.9973	0.0244	0.0504	0:12:47
	4	0.9952	0.0321	0.0510	0.9946	0.0347	0.0591	0:08:32
	5	0.9967	0.0264	0.0476	0.9973	0.0243	0.0501	0:12:05

Table 3: PUB_pept, HBO atomic types

Method	Seed	Training set R^2	Training set RMSD	Training set RMSD$_{at}$	Test set R^2	Test set RMSD	Test set RMSD$_{at}$	Time
EEM	1	0.9962	0.0287	0.0537	0.9963	0.0289	0.0619	0:08:56
	2	0.9961	0.0288	0.0539	0.9962	0.0289	0.0619	0:11:27
	**3**	**0.9963**	**0.0281**	**0.0527**	**0.9964**	**0.0285**	**0.0641**	**0:12:32**
	4	0.9962	0.0285	0.0541	0.9963	0.0286	0.0641	0:11:50
	5	0.9962	0.0286	0.0533	0.9962	0.0289	0.0482	0:11:12
QEq	**1**	**0.9950**	**0.0327**	**0.0742**	**0.9947**	**0.0342**	**0.0747**	**0:15:00**
	2	0.9946	0.0341	0.0760	0.9711	0.0806	0.8585	0:18:03
	3	0.9950	0.0327	0.0741	0.9947	0.0343	0.0746	0:18:08
	4	0.9950	0.0327	0.0742	0.9933	0.0386	0.2144	0:14:56
	5	0.9950	0.0327	0.0742	0.9947	0.0343	0.0745	0:17:12
EQeq	1	0.9961	0.0289	0.0541	0.9962	0.0289	0.0477	0:15:21
	2	0.9962	0.0287	0.0538	0.9963	0.0288	0.0618	0:08:37
	**3**	**0.9962**	**0.0285**	**0.0524**	**0.9963**	**0.0286**	**0.0462**	**0:11:23**
	4	0.9961	0.0288	0.0531	0.9962	0.0289	0.0620	0:12:11
	5	0.9962	0.0286	0.0528	0.9963	0.0286	0.0460	0:13:46
SQE	1	0.9957	0.0303	0.0519	0.9940	0.0363	0.0632	2:05:43
	2	0.9956	0.0309	0.0490	0.9942	0.0359	0.1246	2:20:33
	**3**	**0.9958**	**0.0302**	**0.0510**	**0.9945**	**0.0349**	**0.0608**	**2:28:32**
	4	0.9956	0.0306	0.0495	0.9941	0.0361	0.0583	1:28:59
	5	0.9957	0.0306	0.0525	0.9944	0.0353	0.0630	1:58:56
SQE+q0	**1**	**0.9984**	**0.0188**	**0.0414**	**0.9976**	**0.0228**	**0.2149**	**3:08:40**
	2	0.9982	0.0194	0.0395	0.9982	0.0203	0.0418	2:25:12
	3	0.9982	0.0193	0.0416	0.9978	0.0217	0.05	2:18:27
	4	0.9984	0.0191	0.0404	0.9982	0.0203	0.0436	2:51:13
	5	0.9982	0.0195	0.0419	0.9980	0.0209	0.047	2:50:47
SQE+qp	1	0.9991	0.0136	0.0329	0.9990	0.0146	0.0363	3:05:42
	2	0.9992	0.0133	0.0325	0.9991	0.0143	0.0347	3:23:52
	**3**	**0.9992**	**0.0133**	**0.0322**	**0.9991**	**0.0141**	**0.0342**	**3:08:04**
	4	0.9992	0.0132	0.0323	0.9990	0.0145	0.0410	2:47:15
	5	0.9992	0.0131	0.0324	0.9990	0.0145	0.0365	3:00:07

Table 4: PUB_pept, BA atomic types

3 Correlation graphs from the comparison of empirical charge calculation methods

Following figures show the best results for each seed.

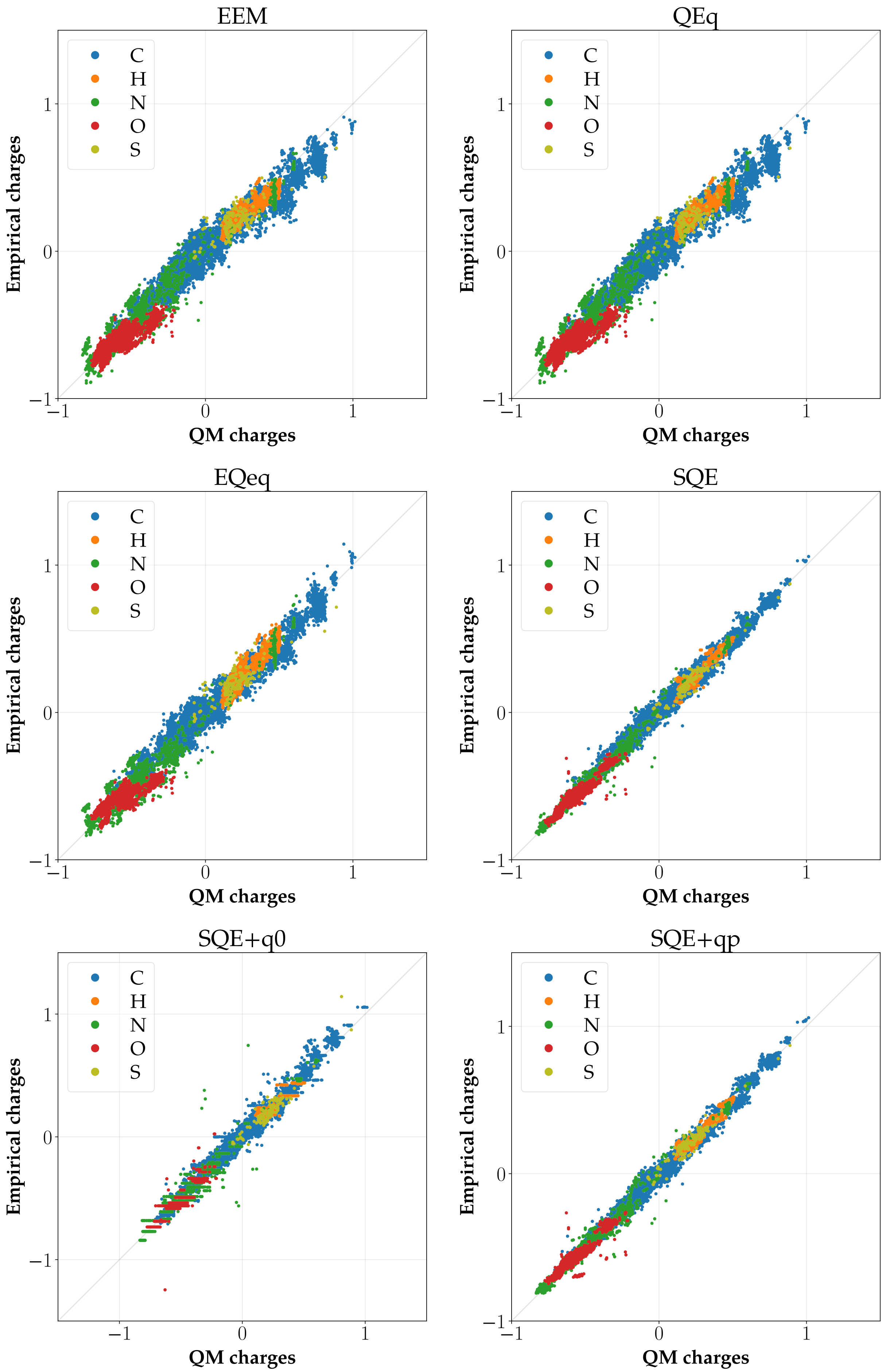

Figure 1: DTP_small

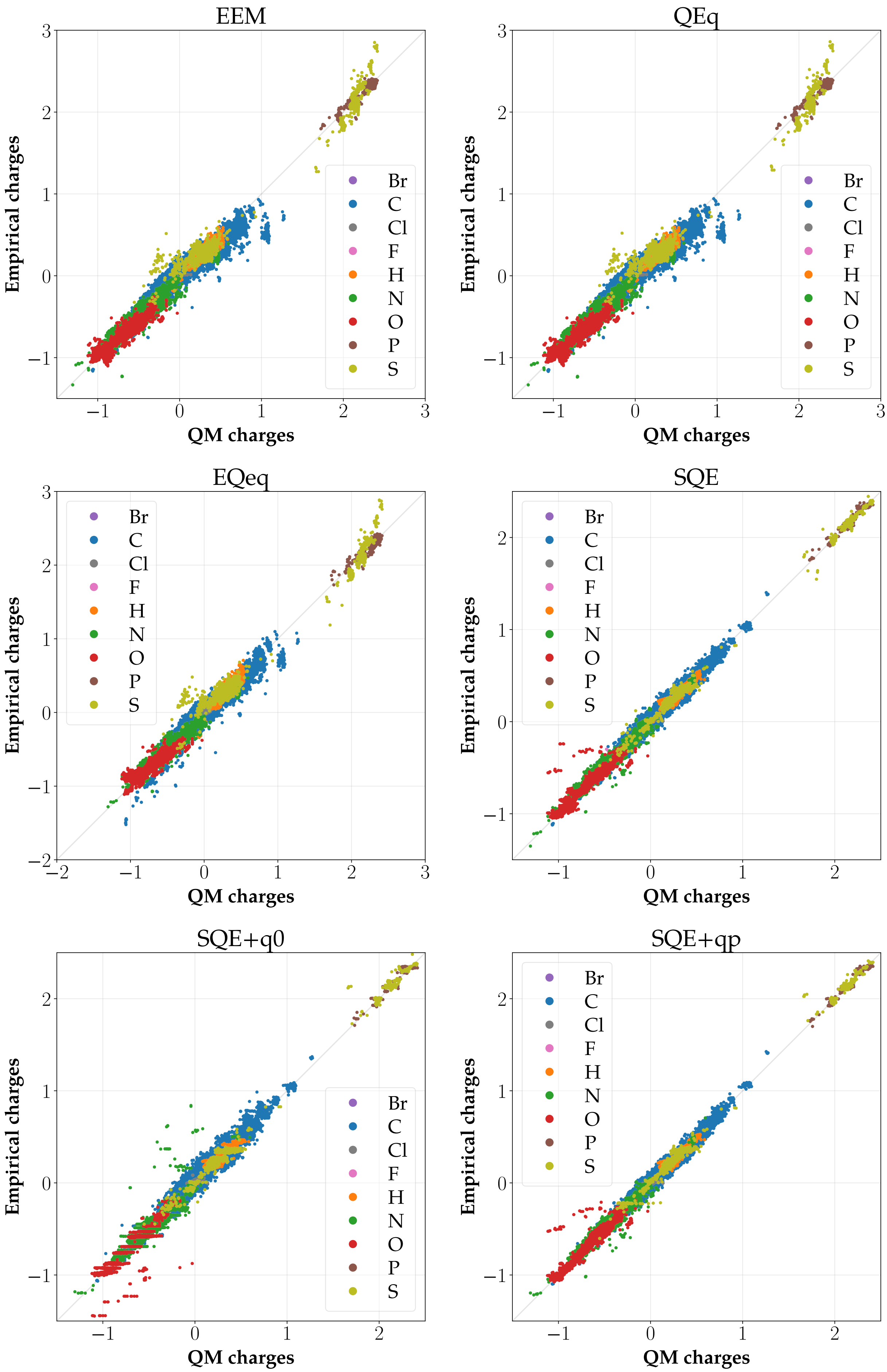

Figure 2: CCD_gen

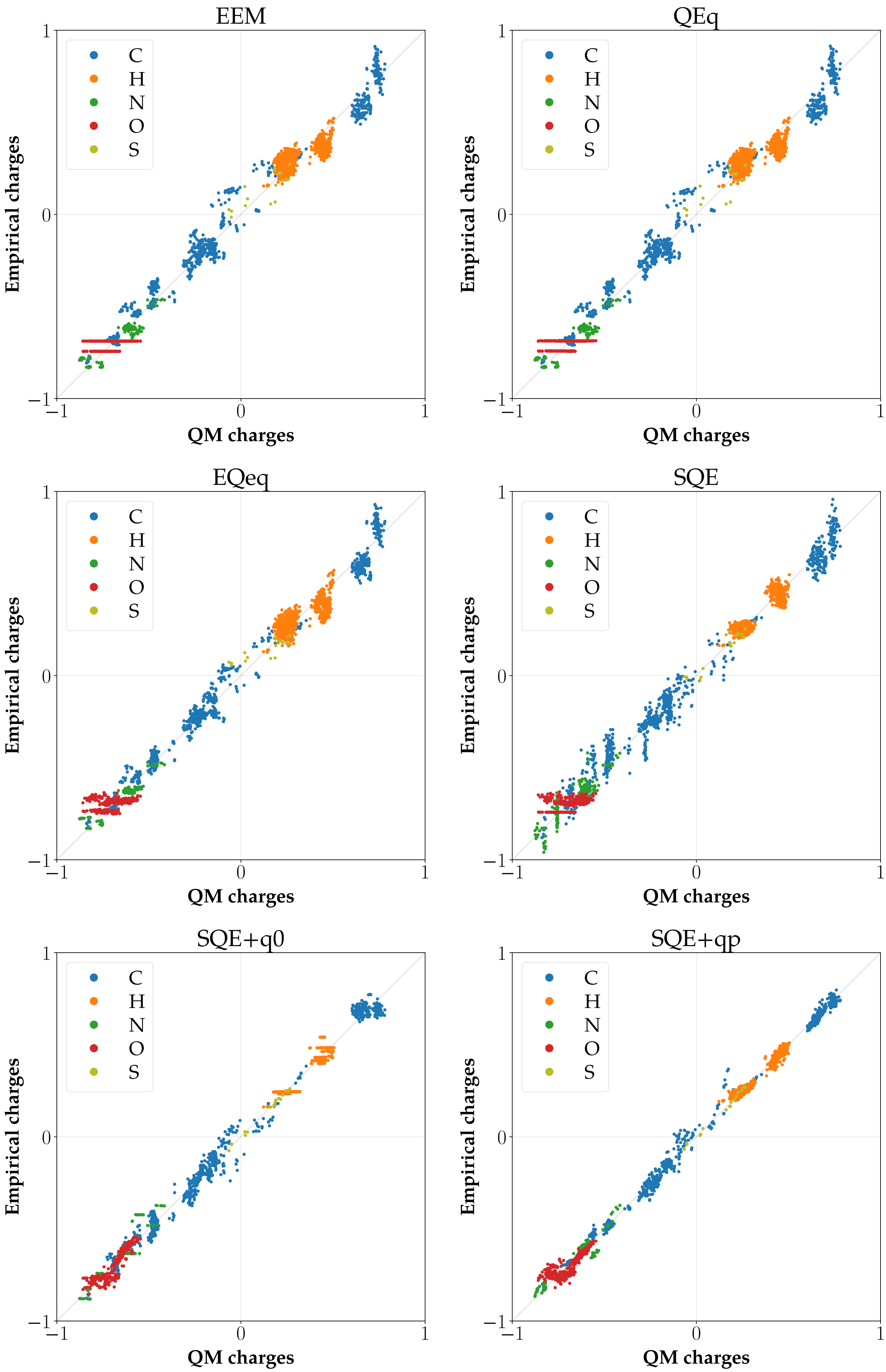

Figure 3: PUB_pept, HBO atomic types

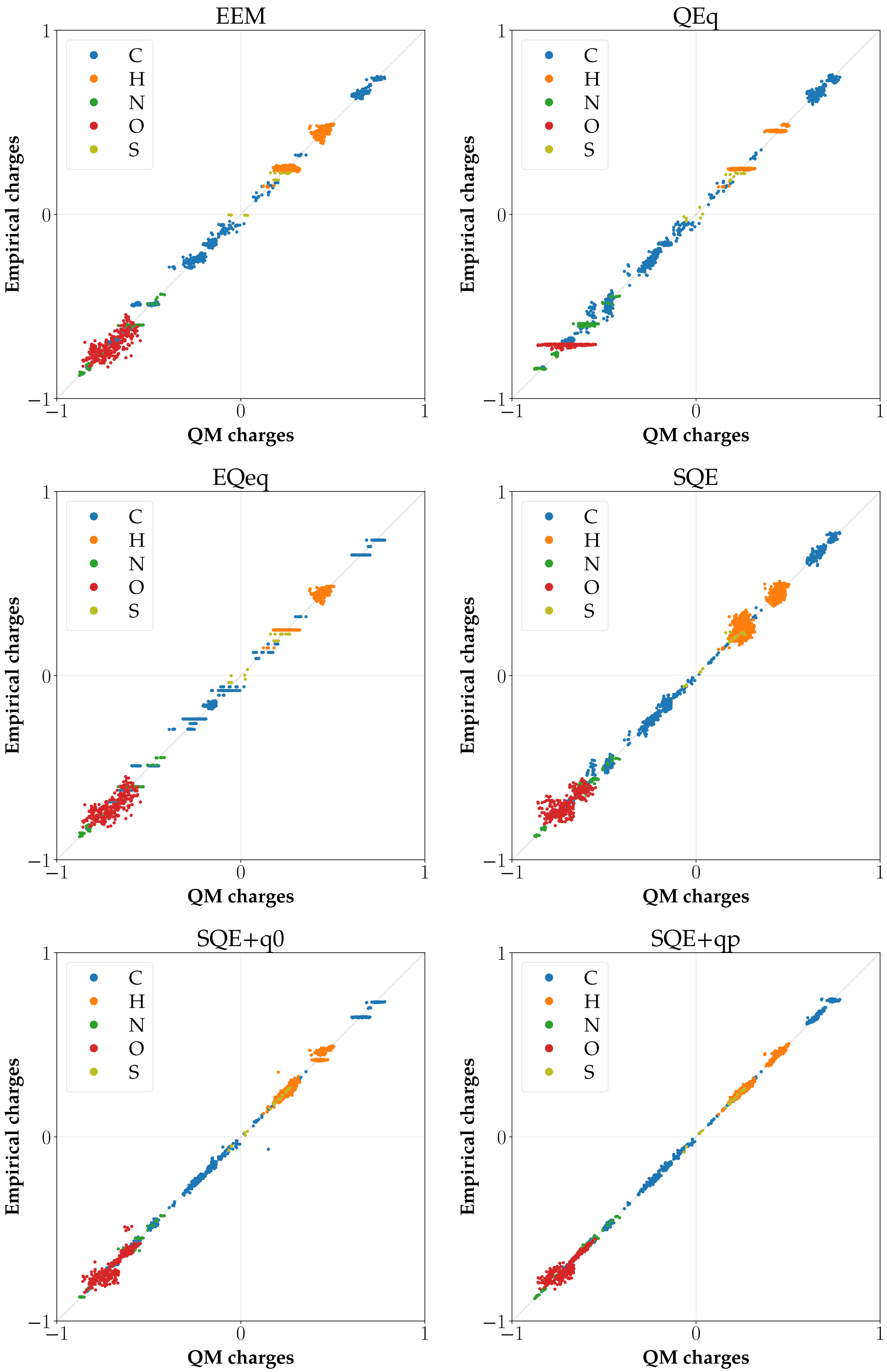

Figure 4: PUB_pept, BA atomic types
